# When Jupiter Meets Saturn: Aby Warburg, Karl Sudhoff, and Astrological Medicine in the Age of Disenchantment

**DOI:** 10.1353/jhi.2024.a926151

**Published:** 2024-04

**Authors:** Xinyi Wen

## Introduction

On 14 January 1927, Fritz Saxl, the deputy director of Kulturwissenschaft Bibliothek Warburg (Warburg Library of Cultural Science), wrote an enthusiastic ten-page letter to Aby Warburg, the eminent art historian and founder of the Warburg Library in Hamburg. In it, Saxl described a research institute with “astonishing similarity [erstaunliche Ähnlichkeit]” to their own library.^1^ Like the Warburg library, this institute was based on an expansive, thematically catalogued collection of books and images. By the time Saxl visited, it already had a collection of over fifteen thousand image plates, mostly photographed historical materials on various subjects, including mythology, religion, astrology, and anatomy. Fascinated, Saxl extended his visit from three to seven days and did not want to leave. He praised the library as “the most interesting and stimulating collection” he had visited in a long time.^2^ This impressive image collection, however, was not part of any art historical institution. It was the Institut für Geschichte der Medizin (Institute for the History of Medicine) founded in Leipzig by Karl Sudhoff, the first professor of medical history in Germany.

This paper examines points of concurrence and divergence in the thought of Aby Warburg and Karl Sudhoff during the 1910s and considers their significance to the histories of science and medicine and the disenchantment thesis. Warburg is widely celebrated today for the novel concepts and methods he contributed to iconology, his *Mnemosyne Atlas*, and the Warburg Library. The past decades have seen an increasing enthusiasm for recovering Warburg’s numerous legacies, accompanied by heated debates and reflections.^3^ While Warburg was mainly regarded as an art historian, his lectures and exhibitions concerning premodern cosmology, anatomy, and healing traditions reflected his deep engagements with the histories of science and medicine.^4^ Warburg’s 1917–18 lectures on astrology in Reformation Germany have been a crucial inspiration for the historical reevaluation of astrology in the twentieth century and remain influential.^5^

To use Sudhoff’s words, he and Warburg were on “parallel journeys,” though their paths sometimes crossed and sometimes diverged.^6^ A founder of modern medical history and an image enthusiast, Sudhoff curated many exhibitions of medical history and compiled several scholarly editions of texts and images of early medicine.^7^ From 1900 through 1915, Sudhoff published several important works on medical astrology and the astrological representation of syphilis in the fifteenth century, which attracted Warburg to join him in curating the Internationale Hygiene-Ausstellung in 1911. As I will show, Warburg’s boxes of index cards reveal how seriously he followed and admired Sudhoff’s works beginning in the 1910s and indicate that Sudhoff’s books were the source of many of the materials he used.

Despite these connections, Warburg and Sudhoff came to very different historical interpretations of the same images. This divergence was rooted in their distinct attitudes toward academic professions and disciplines: while Sudhoff was a dedicated historian of the medical profession and disciplinary leader, Warburg remained an outsider to academia and constantly sought to challenge established disciplinary norms. In the first section, this article considers a brief exchange between Sudhoff and Warbug in 1916 about Saturn’s prosthesis in early astrological prints. This exchange reveals crucial differences in the way they understood images, historical objects, and objectivity, which were strongly shaped by how they understood historical professions and medical epistemologies. The second section turns to their remote collaboration through the executive curator Otto Neustätter, on a twenty thousand-image exhibition, the Internationale Hygiene-Ausstellung in 1911. This collaboration was brought to an unhappy end due to their differing views on the relationship between magic and science, particularly the role of the seemingly “magical” practices in the history of medicine. As we see in the third section, in his 1917–18 Luther lectures on astrological prophecies in Luther’s age, Warburg reinterpreted the very same materials that Sudhoff had studied and developed a fundamentally different theory on the emergence of modern rationality.

Warburg and Sudhoff read one another’s works and exchanged letters between Hamburg and Leipzig, but they never met in person. Even as long-distance communication smoothed the sharper edges of these two distinctive personalities, it nevertheless exposed their crucial differences. The contrasts between Warburg and Sudhoff were intensified when Sudhoff joined the Nazis in 1933, while the Warburg Institute had to be exiled to London. Sudhoff’s sudden turn to the Nazis surprised his fellow medical historians, especially given his previous friendships with Jewish scholars like Warburg. As shown by Vivian Nutton, Thomas Rütten, Dominik Gross, and Werner Friedrich Kümmel among others, Sudhoff’s decision to join the Nazis was a crucial case for understanding the politics of medical-historical research under and after the Nazi regime.^8^

To follow the paths of Warburg and Sudhoff is to understand the entangled relationship between politics, modernity theories, and the histories of magic and science in the twentieth century. Warburg and Sudhoff’s encounter and divergence were embedded in a double context: the theoretical discussions about disenchantment during the First World War and the long historiography of early modern magic and science. On 7 November 1917, five days before Warburg gave his lecture on Luther, Max Weber introduced his famous idea of “disenchantment of the world” in his speech “Wissenschaft als Beruf” (“Science as a Vocation”). Weber’s disenchantment thesis strongly resonated with Warburg’s concerns about the proliferation of superstition in Reformation Germany and during the First World War.^9^ From Weber and Ernst Cassirer to the Frankfurt School, the early twentieth century saw a heated discussion on the emergence of modern rationality, a debate in which Warburg was deeply engaged.^10^

As Dorinda Outram argues, writing the histories of science and Enlightenment has become a way of elucidating the identity of the twentieth century.^11^ Warburg’s textual and institutional legacy has inspired generations of historians to look at early modern occult knowledge in fresh ways and also entered contemporary reflections on the early twentieth-century disenchantment theories.^12^ From Frances Yates and Charles Webster to Brian Copenhaver and Robin Barnes, many historians took Warburg’s Luther lecture as a starting point for reevaluating astrology, medical astrology, and Reformation beyond the disenchantment thesis.^13^ This trend of thought was joined by the emergence of a new social history of medicine in the 1960s and 1970s that inherited Sudhoff’s and his successor Henry Sigerist’s emphases on cultural-historical methods and social contexts. Rather than enhancing Sudhoff’s progressivist narrative, the social-historical approach challenged the established narrative of revolutions and great doctors.^14^ Warburg’s and Sudhoff’s contributions remain relevant to histories of science and medicine, and as we will see, their encounter and divergence in the 1910s still resonate with debates concerning the processes of disenchantment, enchantment, and re-enchantment in the past decades.

## Medical Epistemologies

Let us first turn to 1892, when an epidemic hit Germany. After submitting his doctoral dissertation on Botticelli, the twenty-six-year-old Warburg moved to Berlin and registered for a course in psychology taught by the famous psychologist of memory, Hermann Ebbinghaus, in preparation for systematic training as a doctor.^15^ Warburg’s enthusiasm for a medical career was triggered by the outbreak of a cholera epidemic in his hometown, Hamburg. In a letter to his fiancée, Mary Hertz, in September 1892, Warburg discussed with Mary that he hoped to study medicine so he could serve the city of Hamburg when another epidemic hit; he was frustrated that he couldn’t help at the moment.^16^

At the time Warburg was considering becoming a physician, the professional physician Sudhoff had just turned to historical research. In 1892, Sudhoff moved to Leipzig, bringing with him his collection of books and manuscripts, which he located in a new study in his clinic, next to the consulting room where he met patients. A part-time yet dedicated historian of medicine, Sudhoff usually got up at before 6:00 every morning, if he had not been called to patients at night, and focused on historical work until his morning consulting hours began.^17^ While “visiting his patients on horseback,” in Sigerist’s words, Sudhoff completed many articles and critical editions, including a milestone work: the fourteen-volume complete work of the sixteenth-century Swiss physician Paracelsus.^18^ The everyday balance of medical practice and medical history continued until he became a professor of the history of medicine at the University of Leipzig in 1905, the first chair of the newly established discipline of medical history in Germany.^19^

Both Warburg and Sudhoff came to history through medicine, pursuing their professional identities amid the rapid growth of universities and the institutionalization of art history and medical history in the German Empire.^20^ As Andreas Frewer has shown, Sudhoff was eagerly engaged as a leader in establishing the discipline of medical history.^21^ He regarded medical history as a discipline within the profession of medicine, and his positivist, objective working ethics combined the ethics of historians and physicians, as Claudia Stein has shown.^22^ For Sudhoff, medicine was both his method and his identity. Warburg, on the contrary, was on the marginal end in his discipline. Unable to secure a satisfying university position, he devoted himself to establishing his own Kulturwissenschaftliche Bibliothek Warburg (Warburg Library of Cultural Science), an institution beyond the traditional discipline of art history. The sudden turn from art history to medicine in his youth showed more of his undisciplined nature than his devotion to any discipline.

The question of career paths and disciplinary boundaries emerged in Warburg and Sudhoff’s first conversation. In 1916, the two medically inspired historians directly corresponded for the first time. In this letter, Sudhoff called himself a “specialist historian [of medicine] (Fachhistoriker),” a term he often used to refer to his colleagues and himself. He wrote enthusiastically that “general historians of art [Generalhistoriker der Kunst]” like Warburg could greatly inspire historians of specific professions like himself on their parallel journeys.^23^ In turn, a Fachhistoriker such as himself could also produce valuable resources for art historians. Sudhoff mentioned one of his books, *Beiträge zur Geschichte der Chirurgie im Mittelalter* (Contributions to the History of Surgery in the Middle Ages), an edition of medieval surgical manuscripts containing sixty-five plates.^24^ Warburg actually owned that book.^25^ They both used images from it in the exhibitions they curated. As Matthew Vollgraff has shown, Warburg’s 1927–30 *Mnemosyne Atlas* reused many images from Sudhoff’s earlier display “Drei Jahrtausende Graphik im Dienste der Wissenschaft” (Three Millennia of Graphics in the Service of Science) in the Internationale Ausstellung für Buchgewerbe und Graphik (International Exhibition for the Book Industry and Graphics) in Leipzig, 1914.^26^ While the atlas has been seen as a famously Warburgian method of curating, Sudhoff’s exhibition had already used photographically reproduced images on black panels in the same way as Warburg.

Both Warburg and Sudhoff established their professional expertise, particularly in the study of images, with their expansive collections and detailed studies of primary sources. As Hans Hönes has shown, Warburg’s Hamburgian banker spirit was well reflected in his attempts to gather and sometimes even monopolize materials to guarantee his professional recognition.^27^

A similar story could also be told for Sudhoff’s collection. In Saxl’s words, Sudhoff had an incredible “bomb energy [Bombenenergie]” in expanding his image collection. Spending his funding from Puschmann Foundation “in the most ruthless way,” Sudhoff established a magnificent collection in Leipzig in only a few years.^28^ For Sudhoff, images were irreplaceable “pictorial archives” and “invaluable documents of the theory and practice of our [medical] art.”^29^ Sudhoff earned his professional status through close readings of primary sources, resulting in his numerous short articles and edited volumes. With their collections, Warburg and Sudhoff strengthened their disciplinary expertise and expanded the horizon of art history and medical history with the introduction of traditionally underappreciated visual objects.^30^ Against the background of the “battle of methods,” their approaches were influenced by Karl Lamprecht: Warburg was known as Lamprecht’s disciple, while Sudhoff was sympathetic to “Lamprecht’s call for the introduction of new source material into historical practice both under the subject of cultural history,” despite identifying himself as Rankean.^31^

Technologically, photography enabled their approaches to image collections. According to Horst Bredekamp, Steffen Haug, and Johannes von Müller, the wide application of photography in the early twentieth century was crucial for establishing the approach of “image studies” in its broad sense.^32^ Saxl was very impressed by how Sudhoff’s institute made great use of the only 18×24 camera they had: they photographed numerous images from original manuscripts, stapled photos on boards, bound them into volumes, and shelved them following Sudhoff’s thematic classification system, a process that was similar to Warburg’s method for organizing his photographic collection.^33^ It was in pursuit of reality that Sudhoff turned to photography, the very guarantee of “mechanical objectivity” in his eyes.^34^ For him, any other approach “adds a subjective moment onto the material of investigation and evidence.”^35^ On the contrary, Warburg and his institute used photography as a creative device to duplicate and experiment with images in atlases and archives, as Michael Diers and Katia Mazzucco have shown.^36^

With the “astonishing similarities” in the approaches and contents of their image collections, Warburg and Sudhoff turned their eyes to the same image detail in 1916. But their correspondences exposed their crucial methodological differences. In his first letter to Warburg, following the astrology historian Franz Boll’s introduction, Sudhoff asked Warburg about a specific “image detail [Bild-Detail]”: the personification in astrological iconography of Saturn as a “cripple” using crutches. Sudhoff did not refer to a particular picture, but judging from Boll’s introduction and Warburg’s response, Sudhoff was referring to astrological images and diagrams of planet cycles in premodern Europe. Sudhoff’s interest in this topic was very much a physician’s interest. He noted that he was interested in the prosthesis as a medical historian and also a doctor who had treated crippled legs. As expressed both in his letter and Franz Boll’s letter introducing Sudhoff to Warburg, Sudhoff especially wanted to know when this visual phenomenon first appeared.^37^ Sudhoff was interested in dating this phenomenon probably because it could allow him to locate this iconography in the development of actual prostheses. Representations of medical instruments was the kind of evidence that he always searched for in images of medical history, and he assumed that the image of Saturn’s prosthesis was inherently representational.

In his lengthy reply to Sudhoff on 20 March 1916, Warburg implicitly denied Sudhoff’s assumption that Saturn’s prosthesis represented actual medical practice. He suggested that Saturn’s arm crutch and wooden leg functioned like the scythe in Saturn’s hand—symbols of disability, bondage, and hard labor associated with Saturn, a planet that was known to have negative influence on people since the Hellenistic-Arabic astrological tradition. The prosthesis was only a rough, stylized symbol transmitted across times and places, instead of a vivid representation of the medical practice that could be traced to a specific period or location. To answer Sudhoff’s question about the origins of the prosthesis imagery, Warburg made clear that the earliest Saturn with prosthesis he found was in late fifteenth-century pre-Reformation Germany, in the book of prognostications by court astrologer Johannes Lichtenberger that he happened to be studying for his project on Reformation astrology. He did not mention prostheses or surgery.^38^

While the physician-surgeon Sudhoff naturally saw Saturn’s prosthesis as a treatment or assistive technology for physical disability, Warburg saw it as a symptom of the disabled mind. In his Luther lectures a year later, Warburg argued that early modern people associated Saturn with physical disability because of their long-standing “fear for Saturn [Saturnfürchtigkeit],” a phrase Warburg used to describe their mixed emotion and negative belief toward the planet.^39^ For Warburg, the mentality indicated by images of Saturn’s prosthesis was a real, locatable historical phenomenon, while the assistive instrument it depicted was not. In other words, Warburg analyzed images of bodily disabilities as representations of mental states. As Lucia Ruprecht pointed out, Warburg’s use of medical terms such as symptom, diagnosis, and treatment was primarily in reference to the medicine of the soul, rather than the body.^40^ Warburg’s “historical psychology of human expression” is also a history of mental health as embodied in images, a history of the symptoms and treatments for the “eternally and at all time schizophrenic” human mind.^41^

Notably, both Warburg and Sudhoff approached their images with a medical epistemology. Following Jacob Burckhardt’s famous claim that historical study was “pathological in its kind,” medical concepts such as pathology, symptom, and diagnosis have been frequently adopted as historical methods, especially in Warburg’s works.^42^ As early as 1888, Warburg used medical metaphors to express his epistemological concern for the psychology of expression, resulting in his unique conceptualization of “symptom.”^43^ Following Warburg, Carlo Ginzburg further articulated the paradigmatic significance of medical semiotics for the humanities by pointing out that “clue-hunters” like Giovanni Morelli and Arthur Conan Doyle were originally doctors as well.^44^ Arguably, Sudhoff was another clue-hunter; he poetically compared the vision of artists to the vision of physicians: “The image artist and the true physician have one outstanding property in common, which only they possess in fullest development, namely the single eye to reality … which impels them to pierce all shells, to see the kernel within the shell, the quickening play of life underneath the surface of things.”^45^

Sudhoff’s expression “to see the kernel within the shell” seemed analogical to the Warburgian iconological method: to see visible images as clues for invisible meanings and contexts. For Warburg, the hidden kernel of an image could be energy, pathos, and memory, as Claudia Wedepohl nicely summarizes; for Erwin Panofsky, it was the “intrinsic meaning or content” that “concerns itself with the subject matter of the meaning of works of art.”^46^ Either way, as Emily Levine has shown, the Warburg school suggested the possibility of a “middle ground” between the representational and symbolic, philosophical aspects of image detail, as later developed by Peter Burke.^47^

But this “middle ground” didn’t exist for Sudhoff. For him, the “kernel” was as material as physical anatomy, like the “quickening play of life” of internal organs and the lively interplay of light and shadows in medical scenes. In opposition to a dull, amateur sketch, “the single eye to reality” of great artists allowed them to represent phenomena “wie es eigentlich gewesen [as it actually was],” and “just as true to life and as real in all their details.” Describing artists as “involuntary recorder[s] of reality,” Sudhoff denied the value of artists’ subjectivity and equated images in art with eyewitnessed evidence of historical phenomena. He especially admired Rembrandt’s *Anatomy Lesson* for its lively, realistic style; he saw Rembrandt as a skilled “delineator of reality” for the anatomist and his pupils.^48^ As previously discussed, the shape, form, and use of the prosthesis were on Sudhoff’s mind when he wrote to Warburg. He cared about the medical reality represented by the image rather than the image itself. For Sudhoff, premodern people’s raging fear behind the iconology of Saturn’s wooden legs was nothing but interference with the objectivity of the depicted assistive instruments in the images. The medical epistemology he applied in historical research, or in other words, the vision of “true physicians” he sought after, was a medical gaze onto images, which focused on the images’ representational value for a progressivist history of the medical profession and repressed the emotion they contained.^49^

## A Gallery of Errors

The brief exchange about Saturn’s prosthesis in 1916 was not the first encounter between Warburg and Sudhoff, though it might be the first time they directly exchanged letters. During 1910–11, they had remotely collaborated on the Internationale Hygiene-Ausstellung in Dresden through the executive curator Otto Neustätter, though they failed to meet each other in person. Their divergence is more apparent and unignorable in their curatorial work, which was fraught and ended on an unpleasant note. Despite their shared research interests and familiarity with one another’s works, they had different aims and expectations, particularly as concerned their narratives of the Reformation and the magic-science transition.

In the late 1900s, Sudhoff was invited by Karl August Lingner, a famous pharmaceutical entrepreneur and advocate of social hygiene, to curate the historical and ethnological section of the 1911 Internationale Hygiene-Ausstellung in Dresden. As Stein’s comprehensive study has shown, Sudhoff used his broad network to borrow and buy many images and artifacts. In total, the historical and ethnological section presented 20,394 catalogued items contributed by more than three hundred institutions and individuals.^50^ Many materials from this exhibition, particularly Sudhoff’s historical and ethnological section, were transformed into a permanent display by Lingner after the exhibition and, in 1912, became the foundation of the current Deutsches Hygiene-Museum.^51^

In May 1910, Warburg was invited by Otto Neustätter, executive curator for Sudhoff’s historical and ethnological section, to provide some images representing practices of hygiene in daily life. Neustätter was particularly interested in Warburg’s studies on household items in Renaissance Florence. “We are interested in almost everything intended for daily use, as long as there is some hygienic side to it,” he wrote.^52^ Instead of acceding to Neustätter’s request, Warburg presented several proposals. One proposal offered a particular “paper bread” he acquired from Pueblo Indians during his America trip in 1895; in another, he offered some images of theriac dealers in Renaissance Italy selling the earth of Malta or “St Paul’s earth” as an antidote to snake bites, as Malta was the place where St. Paul performed the miracle of remaining unharmed after being bitten by a snake. Third, Warburg recommended that Albrecht Dürer’s engraving *Melencolia I* should be used as the icon for the whole historical and ethnological section, for it was the ideal representation of early modern psychiatry, in which herbs, amulets, and planetary magical squares were used to cure people’s fear against Saturn. Despite its lack of explicit representations of medicine, Dürer’s *Melencolia I* was highly relevant to hygiene history in Warburg’s eyes: “I do not know a more interesting and beautiful German symbol for the history of the development of hygiene.”^53^

Throughout the curatorial process, Warburg was actively, if not over-actively, involved as one of the three hundred invited contributors, suggesting the exhibition team should emphasize early modern iatro-mathematics, or, in today’s terms, astrological medicine.^54^ He even proposed the curation of a particular section for iatromathematics, on which he was eager to collaborate further with Sudhoff.^55^ Warburg’s enthusiasm was mainly due to his long admiration for Sudhoff’s groundbreaking contribution to the history of astrological medicine. In 1902, Sudhoff had completed one of his most important monographs *Iatromathematiker vornehmlich 15. und 16. Jahrhundert* (Iatromathematicians in the 15th and 16th centuries), which was praised as “the first close and exhaustive study ever made of the true inwardness of medical astrology.”^56^ Warburg’s index cards show that he closely read Sudhoff’s works on astrological medicine. It is from Sudhoff’s *Iatromathematiker* that Warburg learned about the key subject for his Luther lecture astrologer Lucas Gauricus, his dates of birth and death, as well as his 1546 work *Super diebus decretoriis* (On Important Dates). Warburg must have assumed that, by virtue of their shared interests, Sudhoff had a profound understanding of astrological medicine’s importance in developing hygiene.

In his reply to Warburg, Neustätter only expressed interest in the images of theriac dealers in Renaissance Italy.^57^ To elaborate on this proposal, Warburg sent Neustätter a curated display of five images of the sellers of St. Paul’s earth using snakes in marketplace demonstrations in sixteenth- and seventeenth-century Italy, together with six pages of extensive annotations. Warburg called this small display an “episode of the history of magical hygiene [Episode aus der Geschichte der magischen Hygiene]” and a “history of the development of superstitious antidotes [Entwicklungsgeschichte aberglaubischer Gegengiftheilkunde].” As these phrases suggested, Warburg aimed to show that the idea of antitoxic immunization was inseparable from the development of magical beliefs from the Middle Ages to the seventeenth century. Warburg thought this display would interest Sudhoff, the “Historiker der Iatromathematiker,” and enthusiastically looked forward to Sudhoff’s feedback. He mentioned to Neustätter several times that he was very interested in speaking to Sudhoff in person, and, in January 1911, he did travel to Dresden.^58^ He planned to discuss with Sudhoff the materials he contributed and their potential collaboration on the iatromathematical section for the exhibition. Warburg was also keen to visit Sudhoff’s Leipzig library to find more images related to his own research on planetary children, but this visit never happened.^59^ Unfortunately, the two historians missed each other in Dresden after a series of mishaps, including a broken phone at Warburg’s hotel.^60^

In the end, Warburg’s enthusiasm received only a polite but distant reply. Neustätter did not fully understand why Warburg attached such great importance to iatromathematics and only promised that iatro-astrological materials would be in the exhibition, in the sense that “the whole period means an inhibition of hygiene.”^61^ The word “inhibition” precisely depicts Sudhoff’s attitude toward astrological medicine. A phrase Sudhoff often used in *Iatromathematiker* was the “emergence from this sea of error [Meer des Irrthums],” a metaphor describing how “modern medicine” had conquered astrological medicine.^62^ After editing the works of Paracelsus, Sudhoff turned to fifteenth- and sixteenth-century iatromathematics to study the context from which the revolutionary physician Paracelsus emerged, using Paracelsus as a reference point in his historical narrative. In his conference presentation, “Hohenheim und die Medizinische Astrologie (Hohenheim and Medical Astrology),” Sudhoff highly praised Paracelsus’s explicit rejection of astrological medicine. He claimed that this great reformer was “completely free [völlig frei]” from superstition and led the history of medicine onto the right path of observation and experiment.^63^ This image of Paracelsus could be traced back to Heinrich Haeser’s textbook, which Sudhoff used in medical school, a general medical history categorizing Paracelsus as a reformer and the inventor of chemical medicine—a narrative not uncommon in nineteenth-century medical histories.^64^

As if to excuse his hero, when Sudhoff had to talk about Paracelsus’s theory of astral bodies in his lecture, he denied that it was in any way related to astrological superstitions; he read it as a mere metaphor for the essence of natural things. In line with his Paracelsus, Sudhoff kept claiming that while alchemy could be seen as the mother of chemistry and astrology the predecessor of astronomy, astrological medicine, with the connections it drew between the random planetary symbols and the human body, was the least rational of all the types of occult knowledge and completely intolerable for modern professional medicine.^65^

For Sudhoff, exhibiting all the “Irrthurm (Errors)” and “astrologische Irrglaube (astrological superstitions)” in such a large-scale public hygiene exhibition served his pedagogical purposes.^66^ Like Paracelsus, and like the many professional physicians in history who fought against all medical quackery, Sudhoff and Neustätter saw it as their central task to identify and erase superstitious medical beliefs in society.^67^ However, unlike some eighteenth- and nineteenth-century historians who wrote pedagogical medical histories, Sudhoff did not think that a simplistic progressive narrative and judgmental tone were particularly persuasive; his pedagogical and scholarly principles were coherent with Rankean empiricism.^68^ He believed that objectively and impartially presenting “a gallery of charlatans” was a way of fighting against quackery, as it could help people distinguish errors and superstitions from correct modern practices.

Although Neustätter thankfully accepted Warburg’s extensively annotated display, when Warburg went to see Sudhoff’s exhibition in person, he found only one of his five images of Renaissance Italian theriac dealers exhibited. Even worse, it was mislabeled both in the exhibition and in the catalogue—”Paulus” miswritten as “Petrus.”^69^ In the summer of 1911, Warburg sent several angry letters to Neustätter asking for an explanation, and he even planned to retract his contributed materials.^70^ After Neustätter promised to correct the mistake about St. Paul, Warburg finally accepted Neustätter’s apology.^71^ However, Neustätter’s diplomatic apology did not mention their deeper rationale. Warburg’s contribution was considered nothing more than a direct representation of medical practice. Neustätter considered Warburg’s images to be placeholders for representing premodern mithridatism (that is, administering small doses of toxin for immunization), barring that they could not find better representative images.^72^

Nearly twenty years later, Sudhoff spoke proudly of this exhibition in his memoir. He considered this exhibition an unparalleled success in his life: “how fully developed the path was, starting with food, food preparation, kitchen equipment, … with every detail, from the roof to the toilet room in the house.”^73^ While Sudhoff was proud of the scale of his exhibition, Warburg was angry over the tiniest mistake on the tag of a single image. This little mistake was of no concern to Sudhoff: no matter whether it was St. Paul or St. Peter, what mattered was that the image showed a man selling theriac to represent the antitoxic practice. However, for Warburg, the iconological connection between the snake-bitten St. Paul and the snake rod of the medical god Asclepius was a crucial element in the images. What guaranteed the antitoxic effect was not material ingredients but rather the immaterial wonder performed by St. Paul and the long-standing symbolism of snakes in medicine. In Warburg’s display, the snake-waving theriac dealers were not simply practitioners of mithridatism but also carriers who revived and transmitted the symbolic meaning of the snake.

In panel 28/29 of *Mnemosyne Atlas*, Warburg used the same images he sent Sudhoff, which illustrated the revival of ancient beliefs in Renaissance Italy.^74^ While “a historical progression toward greater enlightenment may plausibly be ascribed to these panels,” as Christopher D. Johnson points out, Warburg’s *Mnemosyne Atlas* did not use them to indicate the difference between premodern medical superstitions and modern rational medicine, as Sudhoff saw them.^75^ Rather, Warburg’s “episode of the history of magical hygiene” demonstrated the entangled relationship between magic and science. To use Gertrud Bing’s words, it showed how immunization, one of the key concepts in modern medical sciences, “comes not from the traditional lineage of reason, but rather from monstrous-causal thought.”^76^

## Regressive Disenchantment

Despite their curatorial divergence, neither Warburg nor Sudhoff was satisfied with a simplistic narrative of progression, and neither of them ever abandoned the idea of progression. Their divergence was not a simple conflict between Whiggish history and anti-progressivism but a more subtle and sophisticated disagreement about how historical changes should be addressed in temporal frameworks. This section will focus on how Warburg and Sudhoff studied the very same material of Reformation astrological medicine and how they developed different historical evaluations of astrological medicine along the way. Sudhoff maintained his judgment of astrological medicine as “superstition” balanced with an awareness of its social contexts, while Warburg developed a theory of regressive progression in which astrological medicine played a crucial role in the emergence of modern rationality.

As Stein has shown, Sudhoff was committed to the Rankean principle of measuring the past by its own standards.^77^ This historicism allowed Sudhoff to keep a performative distance from existing progressive, pedagogically designed narratives of medical history and present himself as the more objective and sympathetic narrator. He once encouraged his readers to be more understanding and less judgmental of the medical astrologists, for those practitioners “did not have teachers like Vesalius or Kepler.”^78^ As shown in the earlier section, Sudhoff was not always sympathetic to these “least rational and completely intolerable” astrologists. But when interpreting specific historical sources, he always tried to make sense of medical astrology in its social context.

In his edited volumes of early syphilis literature (1912), Sudhoff pointed out that when syphilis was raging in Europe in 1484, it was astrologists who first recorded the symptoms of syphilis in their pamphlets, for they took it as the “plague” caused by the great conjunction of Jupiter and Saturn on November 25, 1484. Sudhoff regarded the astrological explanation of syphilis as “Irrglaube” but acknowledged that these astrological fantasies contained “everything that first came to light about the ‘new illness’” and had great value as historical records.^79^ One example is *Prognostica*, a pamphlet written by astrologer Paulus von Middelburg containing a set of astrological prophecies on the disastrous consequences of the Jupiter-Saturn conjunction in 1484.^80^ Sudhoff evaluated its function as twofold: First, it was the first printed account of syphilis; second, its popularity allowed doctors from remote areas to know about this new epidemic and diagnose patients accordingly.^81^ As a cultural historian of medicine, Sudhoff gave a functional explanation of the role of astrological medicine in an epidemic. He maintained his initial judgments of astrological medicine but was also able to understand its practical value at that time.

This reproduction of von Middelburg’s *Prognostica*, included in Sudhoff’s *Syphilisliteratur*, later became a crucial source for Warburg’s Luther lectures. In his index cards, Warburg sketched out his most important takeaways from *Syphilisliteratur*: one is the woodcut image on the title page of *Prognostica*, an image depicting Jupiter (the monk) and Saturn (the farmer) under the constellation of Mars; the other is Dürer’s *Syphilitic Man*. It was Sudhoff who first pointed out the relation between the two images: this plain, scrawly illustration in *Prognostica* could be the source of Dürer’s *Syphilitic Man*. In *Syphilitic Man*, the celestial sphere above the man’s head, with twelve constellations and the year 1484 in its center, is referred to as the great conjunction. Above and below the middle number 4, the position of Scorpio and Aries indicated the location where Jupiter and Saturn conjuncted on 25 November, as Warburg emphasized in his sketch. The fact that Dürer referred to the astrological explanation of this disease as having originated from von Middelburg in his depiction of syphilis was significant for Warburg’s ongoing work on Luther and Dürer. In May 1917, Warburg wrote to Sudhoff to express his appreciation for this source and asked for more photographs of *Prognostica*.^82^ Later in his Luther lectures, Warburg thanked Sudhoff for this groundbreaking discovery of the relation between Dürer and popular astrological medicine.^83^

However, Warburg had a greater ambition. Upon receipt of Sudhoff’s photographs of *Prognostica*, Warburg told Sudhoff that he proved a “very remarkable relation” between Paulus von Middelburg and Johannes Lichtenberger, court astrologer of Friedrich III. He discovered that Lichtenberger’s *Prognosticatio*, a popular prophecy book prefaced by Luther, was plagiarized mainly from von Middelburg’s *Prognostica*.^84^ For Warburg, this discovery connected Luther’s and Dürer’s views on astrology: they were both influenced by the widely circulated beliefs on the horrible consequences of the 1484 Jupiter-Saturn conjunction. In his letter to Sudhoff, Warburg confidently said that this discovery would “yield desirable and indisputable enlightenments for the evaluation of the earlier *Prognostica* from the perspective of general cultural science.”^85^ Implicitly, this meant doing away with Sudhoff’s functional evaluation under his progressive framework.

A year later, in his lectures, based on Luther’s preface to Lichtenberger’s *Prognosticatio*, Warburg demonstrated an important paradox behind Luther’s hostility to astrology: despite having harshly criticized astrology, Luther was not “completely free [völlig frei]”—using the exact phrase that Sudhoff used to describe Paracelsus—from astrological belief. Like Paracelsus, Luther considered astrological reasoning as irrational and casual in general. Still, when it came to Jupiter and Saturn’s conjunction, he nonetheless believed in Lichtenberger’s (and, originally, von Middelburg’s) prediction of all those natural disasters and plagues. Luther regarded astrological events and natural disasters as meaningful messages from God and his angels, and in his eyes, such a significant Jupiter-Saturn conjunction must have its indications.^86^ Warburg pointed out that despite being the pioneer of the Reformation, Luther was not free from the enchanted world; he feared the greater supernatural power behind natural phenomena. This argument profoundly influenced later historiography: following Warburg, historians such as Charles Webster argued that this paradoxical attitude was also found in other Protestant scholars, including Kepler, “the Luther of astronomy,” and Paracelsus “the medical Luther,” the latter having commented on the same prophecy book by Lichtenberger.^87^ In Warburg’s words, they rejected astrology because of its ways of reasoning, not because of its deterministic premise of incomprehensible supernatural power over humans.

However, Dürer offered a different solution. Dürer’s *Syphilitic Man* was also inspired by von Middelburg’s astrological prediction, but Warburg pointed out that *Syphilitic Man* was motivated by Dürer’s scientific interest in this physical phenomenon rather than any fear toward the omen itself. This argument sounds more convincing in light of recent studies showing that Dürer’s “syphilitic man” could be the artist himself, who was then suffering from the disease. In this case, his woodcut could be seen as an attempt at recording, understanding, and healing.^88^ In *Melencolia I*, healing became Dürer’s own way of neutralizing the fear of great conjunction. While the crippled-leg Saturn in German iconological tradition symbolized illness and the cause of melancholy, Dürer turned Saturn into a contemplating healer and creator. He replaced Saturn’s crutch with a compass, the symbol of rational creativity, and equipped her with the teukrion, a traditional herb against melancholy. The great conjunction was implied by Jupiter’s magical square on the wall behind Saturn, an object traditionally used as an iatromathematical remedy against melancholy, according to Ficino. In this way, Saturn’s contemplative, self-healing effort turned a disastrous conjunction into a therapy that utilized Jupiter to neutralize Saturn’s negative effect. To Warburg, this astrological remedy for melancholy affirmed a human’s creative ability to reverse the dreadful consequence implied by old astrological symbolism through healing.

Warburg was fully aware that Dürer’s therapy in *Melencolia I* was not simply invented by a genius artist but originated from the tradition of astrological medicine, the “least rational” medicine in Sudhoff’s eyes. Based on planetary sympathy and antipathy, it has long been a healing principle in astrological medicine to use herbs and artifacts signifying Jupiter against diseases caused by Saturn. When preparing for his Luther lectures, Warburg kept searching for more materials on remedies using Jupiter against Saturn. From Sudhoff’s *Syphilisliteratur*, Warburg copied on his index card an excerpt of a poem by Sebastian Brant demonstrating how Jupiter expelled the melancholy brought by Saturn and later specifically asked Sudhoff for more materials on “iatromathematical remedies against Saturn’s influence.”^89^ In this way, *Melencolia I* also embodied the various astrological remedies in Dürer’s time. Warburg argued that Dürer was not trying to reject astrology entirely but to modify and remedy it within the astrological tradition and bring out its intrinsic rational side. In Ernst Cassirer’s words, Warburg was trying to show that astrology “presents a double intellectual front.”^90^

From Paracelsus and Luther to Dürer, from Sudhoff to Warburg, we can recognize two stories of the emergence of modern rationality. While the first story depicted a new rationality winning against the “superstitious” astrological medicine, the second story allowed it to lose the battle, and considered losing necessary. In the first story, rationality was established through academic debates and pamphlet wars that promised a complete victory of the rational side. From this perspective, the visual language of Dürer’s *Melencolia I* was still highly emblematic and mystifying, which suggested this image’s failure to undo the irrational association between planets and people. But in the second story, establishing rationality required a surrender, by acknowledging and building on what was considered superstition. By not abandoning but entering this system of astrological association, Dürer and Brant turned this long-standing association into nothing but a disease that people could counter with remedies, and turned the fear against Saturn into something curable. In contrast, the so-called “victory” through simply declaring astrology irrational was actually a failure to act upon this belief in a rational and effective way. In the second story, the emergence of modern rationality does not require a complete disenchantment of the world. Instead, human beings become truly rational by entering and facing the enchanted world, and asserting their own will in the process. Iatromathematics, or astrological medicine, played two different roles in these two stories. Whereas for Sudhoff, astrological medicine was the least rational among all occult arts, for Warburg, it contained the foundation of rationality. The true value of Dürer’s astrological remedy did not lie in treating physical diseases, but in healing human souls repressed by either godly or demonic superior powers.

Decades later, Warburg’s story was further developed by the intellectual historian Frances Yates, who ascribed astral medicine as a “scientific escape from determinism.”^91^ Despite the controversies that emerged through the debates going on since the 1970s concerning the “Yates thesis,” as summarized by Yates’s opponents, the importance of occult knowledge for seventeenth-century science has been generally recognized today.^92^ Meanwhile, the debate between Hans Jonas and Hans Blumenberg suggested a possible danger behind Warburg’s story. While Warburg avoided putting astrological medicine under the magic-science dichotomy by focusing on its value for human beings’ autonomy and liberation from fear and determinism, this might have created another dichotomy between repression and liberation, which “had come to replicate gnostic dualism.”^93^

Magic versus mathematics, logic and science, superstition versus rationality, these pairs of concepts constituted the polarities Warburg sought in the “eternally schizophrenic” Western minds. Early modern European astrological medicine embodied this eternal struggle in a particular historical time. The words “magic” and “astrology” in Warburg’s writings were used with variations and nuances. To some extent, they were both historical and ahistorical concepts: they sometimes referred to historically concrete disciplines or practices, as used by his contemporary historians like Boll and Sudhoff. Sometimes, mainly when used as adjectives, they referred in a psychological sense to certain “superstitious—theoretical or practical—associations between man and object” that could exist in any time and practice, as influenced by anthropologists such as Edward Tylor.^94^ What Warburg called superstition was both an eternal psychological condition of human beings, and a phenomenon in various historical and cultural contexts that drives people like Dürer into the process of becoming rational, in different times and places and in their own ways. Meanwhile, despite Warburg’s emphasis on continuities and repetitive polar movements, his oeuvre never entirely excluded revolutions and progressions. As Horst Bredekamp and Claudia Wedepohl have shown, Warburg also argued that the eclipse orbit of Mars calculated by Kepler suggested a profound departure from the mystical projection of a round celestial sphere onto heaven.^95^ But, contrary to Sudhoff, Warburg saw the existence of revolutionary figures or movements more as abrupt, significant symptoms of their underlying polar dynamics than as decisive forces themselves. To Warburg, the significance of Dürer’s astrological medicine did not lie in its teleological relationship with the future but in its restorative relationship with the past. As Spyros Papapetros argues, in the 1920s, Warburg and Saxl developed the idea of “regressive evolution [rückfällige Entwicklung]”to describe how ancient cosmological images that were previously treated as mere metaphors were revived and put into practical application in late medieval and early modern times.^96^ In this way, Dürer’s reinterpretation of the ancient symbolism of the Jupiter-Saturn conjunction was also a revival of metaphors with the practice of herbal remedies. The mode of transition Warburg sought in his Luther lecture can be seen as a regressive evolution carried out by acknowledging the existence of old beliefs and reviving them with reason and practice. In the regressive model of transition, it is essential to revive the old form of astrological symbolism in order to reinterpret and act upon them, avoiding the irrational illusion of complete victory. The rationalization is always an unfinished project—and, paradoxically, only unfinished projects can continue to progress.

## Conclusion

In light of ongoing debates concerning disenchantment, this article has argued that the encounter of Warburg and Sudhoff resulted in a clash of two opposing historical temporalities and generated a different understanding of the early modern transition beyond the progressive discourse. Their encounter and divergence did not emerge from theoretical criticisms of modernity but from sorting pieces of historical materials with boxes and plates. The early twentieth-century institutionalization of humanities disciplines and the growing institutional collections of historical materials fundamentally shaped both Warburg’s and Sudhoff’s historical interpretations. As Lauren Kassell pointed out, the culture of collection and the rising antiquarianism have disenchanted surviving magical objects since the eighteenth century.^97^ Guided by his Rankean positivism, cultural-historical ambition, and dedication to a medical history that serves the medical profession, Sudhoff disenchanted the objects he collected by reducing them to their medical and hygienic functions and disregarding the beliefs and emotions behind them. Using Nietzsche’s words, Sudhoff’s collection of medical objects suffered from the illness of historicism: preserving everything from the past as a gesture of breaking free from the past.^98^

Yet Warburg re-enchanted the same objects that were disenchanted by Sudhoff. By acknowledging symbols, gestures, and mentalities as objects for history, Warburg identified the surviving magical beliefs in what was understood to be purified modern medical practice. He acknowledged the co-existence of old symbolism and new ideas and practices captured in images and refused to depict the emergence of modern rationality as a radical rejection of the past. Instead, he depicted it as a regressive progression, where modern people established their rationality and autonomy through reengaging with the enchanted symbolism from the past and turning the determined into something operable. As Bruno Latour pointed out, as modernization created the purified, separated domains of nature and culture, it also led to a historical narrative claiming a dualist separation between past and present.^99^ Whereas Sudhoff defended a medical modernization that has eliminated other “old” and “superstitious” practices, Warburg showed us a modernization that was conditioned by the hybridity of healing practices and the coexistence of different temporalities. Despite that, this encounter was not Warburg’s revolution against the progressive histories of science represented by Sudhoff. Instead, it was also a regressive progression in which Warburg integrated the ideas of progress and revolution in Sudhoff’s sense into his narrative of the emergence of modern reason.

Facing the survival of astrological medicine in the modern age, Warburg and Sudhoff’s debate over the Jupiter-Saturn conjunction remains relevant to debates on the idea of disenchantment up until today. As Jane Newman and Gottfried Korff have shown, Warburg turned to the study of Reformation astrology in search of a solution for the raging superstitions during the First World War.^100^ As Warburg filled his index card boxes with numerous newspaper clips of war-related superstitions, Sudhoff sought to institutionalize medical history to prosecute the “ever-increasing inroads of quackery” in his time.^101^ Like Warburg and Sudhoff, Weber was also well aware of his contemporary practices of mysticism and even believed that he “must experience these psychological states to understand” the historical meaning of mysticism.^102^ In other words, major twentieth-century thinkers who contributed to the theorization of disenchantment were aware that their worlds had never been entirely disenchanted. As shown by Wouter J. Hanegraaff, Egil Asprem, and Jason Josephson-Storm, there was a strong connection between interwar occult cultures and the emergence of disenchantment discourse during this period.^103^ Under the rising awareness of the surviving magical practices in modern society and the historical re-evaluation of premodern occult sciences since the 1970s, re-enchantment in the Enlightenment and the industrial age has been heatedly discussed over the past twenty years.^104^ These debates have had large ripples. As Alexandra Walsham has pointed out, this recent historiographical awareness of the circular movements of re-enchantment and disenchantment counter-acts the dangers of progressivist narratives and linear developments.^105^ While the dynamics of disenchantment, enchantment, and re-enchantment were often thought to have entered historiographical discussions only recently, Sudhoff and Warburg already explored these issues in the clash of their notions of progressive and regressive disenchantment. The idea of a regressive, recurring, and never-ending disenchantment has been raised to overcome the linear narrative of progress, concurrently with the birth of the disenchantment discourse in interwar Germany.

University of Cambridge.

## Figures and Tables

**Fig. 1:**
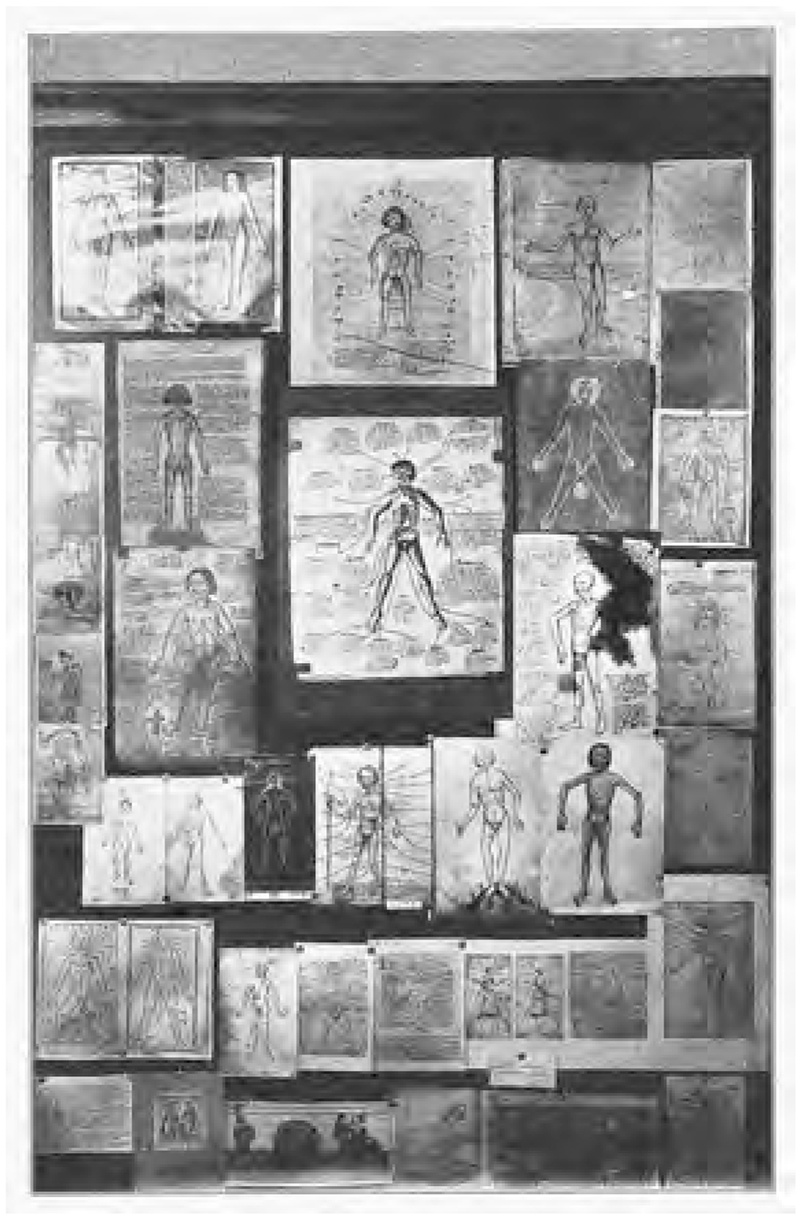
Panel of anatomical images and the Zodiac Man, curated by Karl Sudhoff, Internationale Ausstellung für Buchgewerbe und Graphik 1914 in Leipzig. In *Archiv für Buchgewerbe*, vol. 51 (Leipzig: Verlag des Deutschen Buchgewerbevereins, 1914), Tafel XI. Internet Archive, Public Domain.

**Fig. 2:**
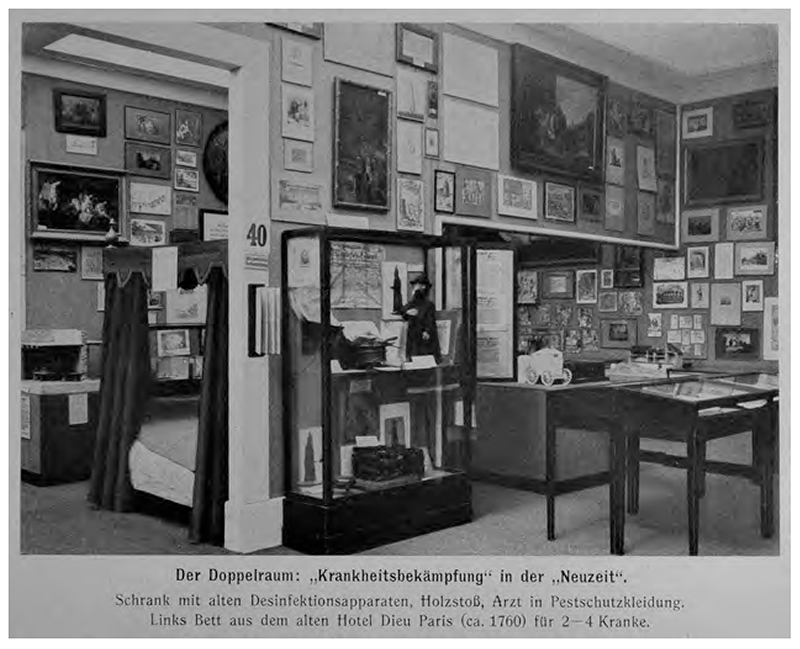
Photo of the the 1911 International Hygiene Ausstellung. From *Historische Abteilung Mit Ethnographischer Unterabteilung. I: Historische Abteilung. 2. verb. und illustrierte Aufl*. (Dresden: Internationalen Hygiene-Ausstellung, 1911), 401. Wellcome Library, CC BY 4.0.

**Fig. 3:**
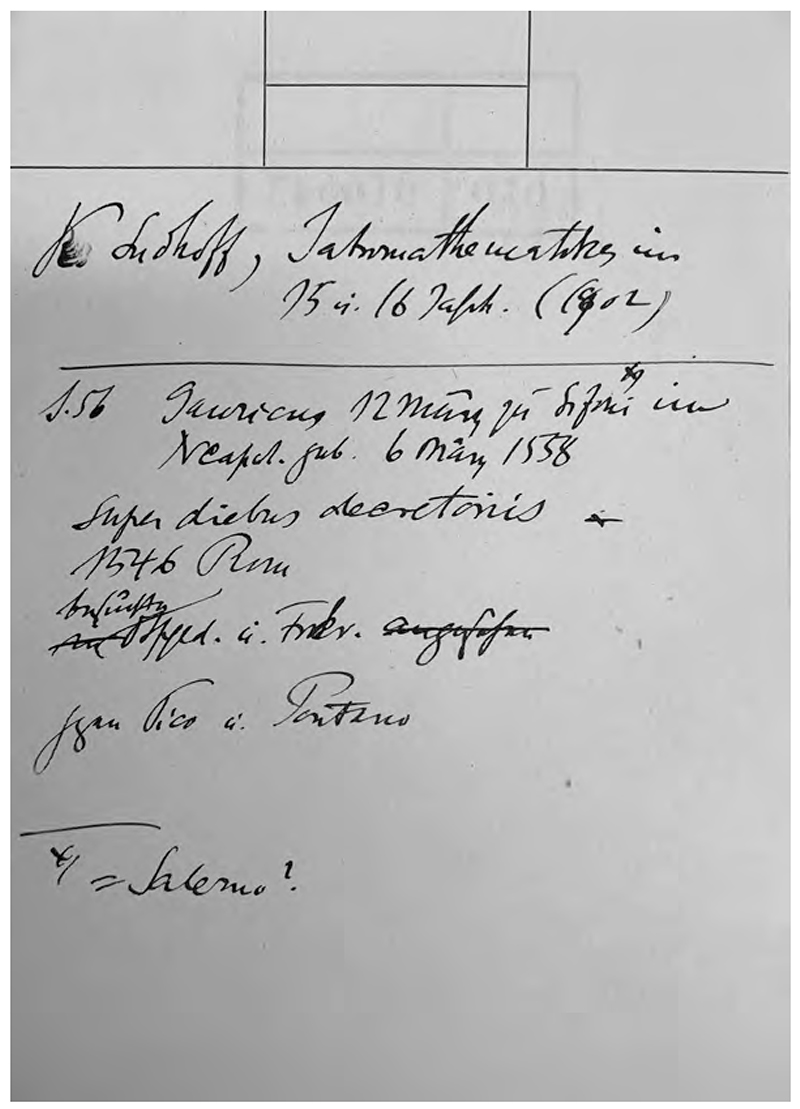
Warburg’s index card recording bibliographical information about Lucas Gauricus, adapted from Karl Sudhoff, *Iatromathematiker vornehmlich im 15. und 16. Jahrhundert* (Breslau: J.U. Kern & M. Müller), 1902). Warburg Institute Archive, Zettelkästen 30, 016043. Copyright: The Warburg Institute.

**Fig. 4:**
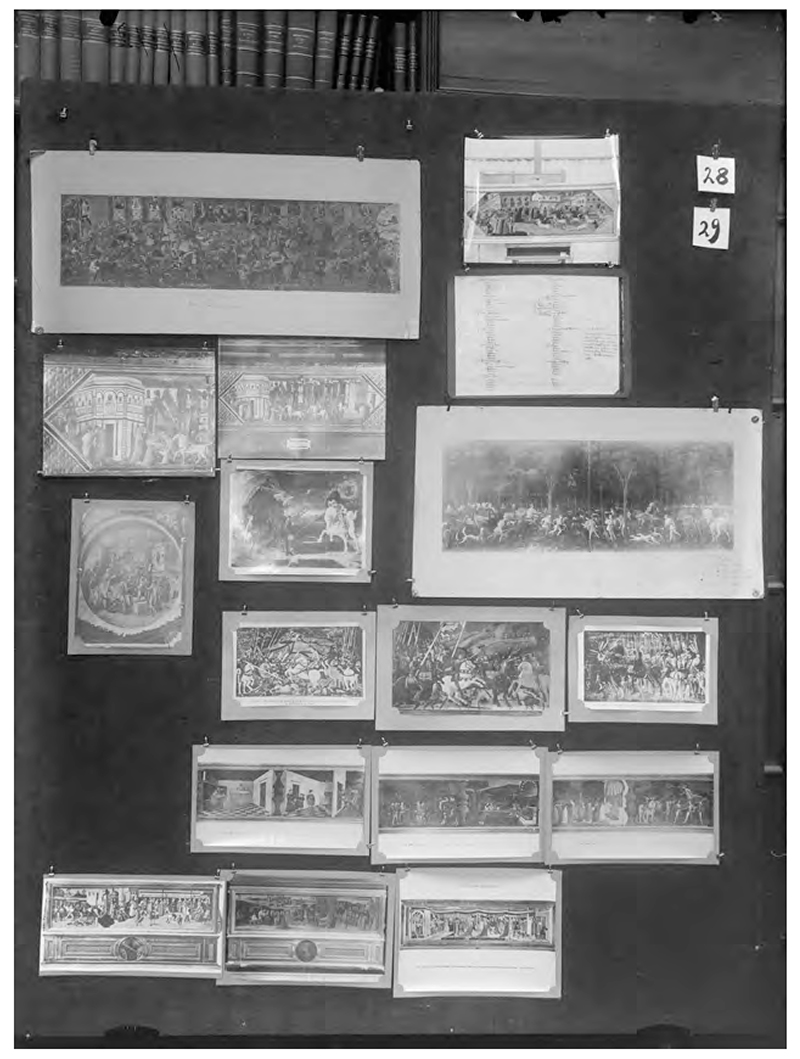
Aby Warburg, *Mnemosyne Atlas* (1929), Panel 28/29. In this panel, two images on the second row of the top left area (*Fest des hl. Johannes mit Theriakverkäufer*, Cassone, Florence, the first third of 15th century, Florence, Museo Nazionale del Bargello), and the image below them on the left (*Heilmittelverkäufer (Theriakverkäufer)*, Agostino da Mozzanega, Fresko, around 1528, Mantua, Palazzo del Te, Camera dei Venti), were recycled from the display Warburg curated for Sudhoff’s exhibition. Copyright: The Warburg Institute.

**Fig. 5:**
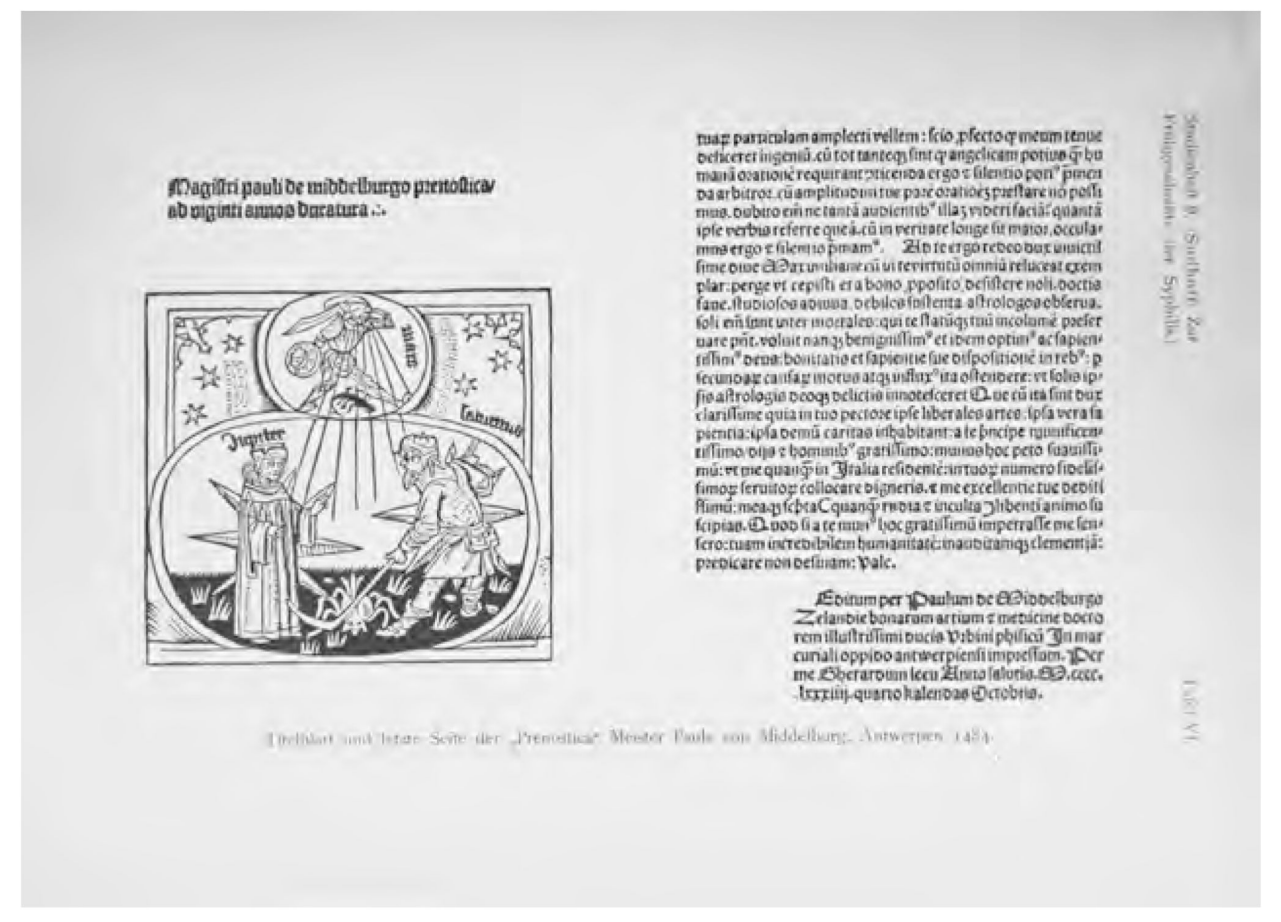
Title image of Paulus von Middelburg, *Prenostica ad viginti annos duratura: Mit widmungsbrief des autors an Maximilian, Erzherzog Von Österreich, Löwen 31. 8. 1484* (Leipzig: Martin Landsberg, 1484). From Karl Sudhoff, *Aus der Frühgeschichte der Syphilis; Handschriftenund Inkunabelstudien, epidemiologische Untersuchung und kritische Gänge* (Leipzig J. A. Barth, 1912), Tafel VI. Internet Archive, Public Domain.

**Fig. 6:**
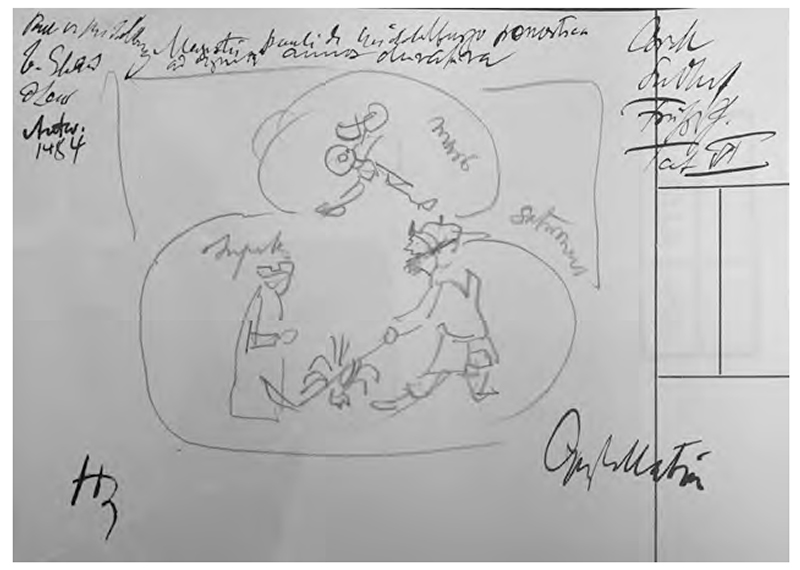
Warburg’s index card sketch reproducing the title image of *Paulus von Middelburg, Prenostica ad viginti annos duratura: Mit widmungsbrief des autors an Maximilian, Erzherzog Von Österreich, Löwen 31. 8. 1484* (Leipzig: Martin Landsberg, 1484), from *Karl Sudhoff, Aus der Frühgeschichte der Syphilis; Handschriftenund Inkunabelstudien, epidemiologische Untersuchung und kritische Gänge* (Leipzig J.A. Barth, 1912), Tafel VI. Warburg Institute Archive, Zettelkästen 29, 016138. Copyright: The Warburg Institute.

**Fig. 7:**
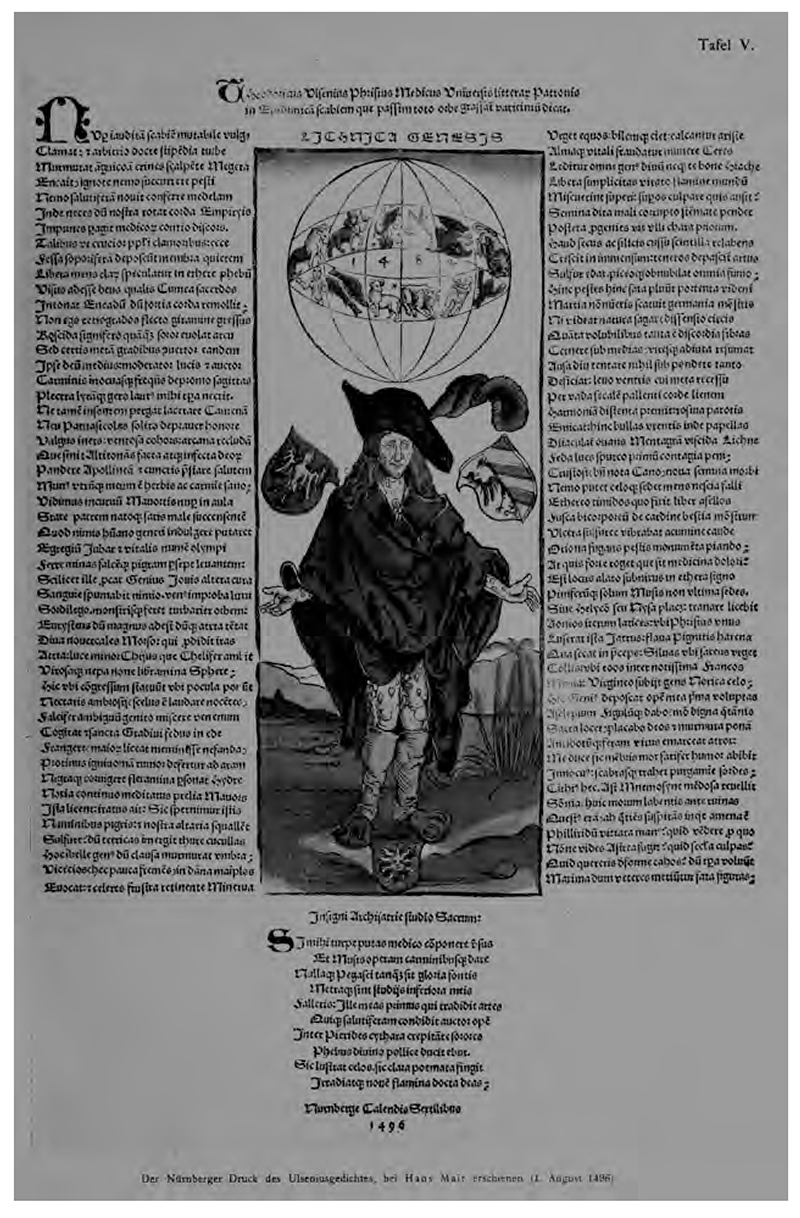
Albrecht Dürer, *The Syphilitic Man* (Nürnberg: Hans Meier, 1496). From Karl Sudhoff, *Graphische und typographische Erstlinge der Syphilis-literatur aus den Jahren 1495 und 1496* (München: Carl Kühn, 1912), Tafel V. Wellcome Library, Public Domain.

**Fig. 8:**
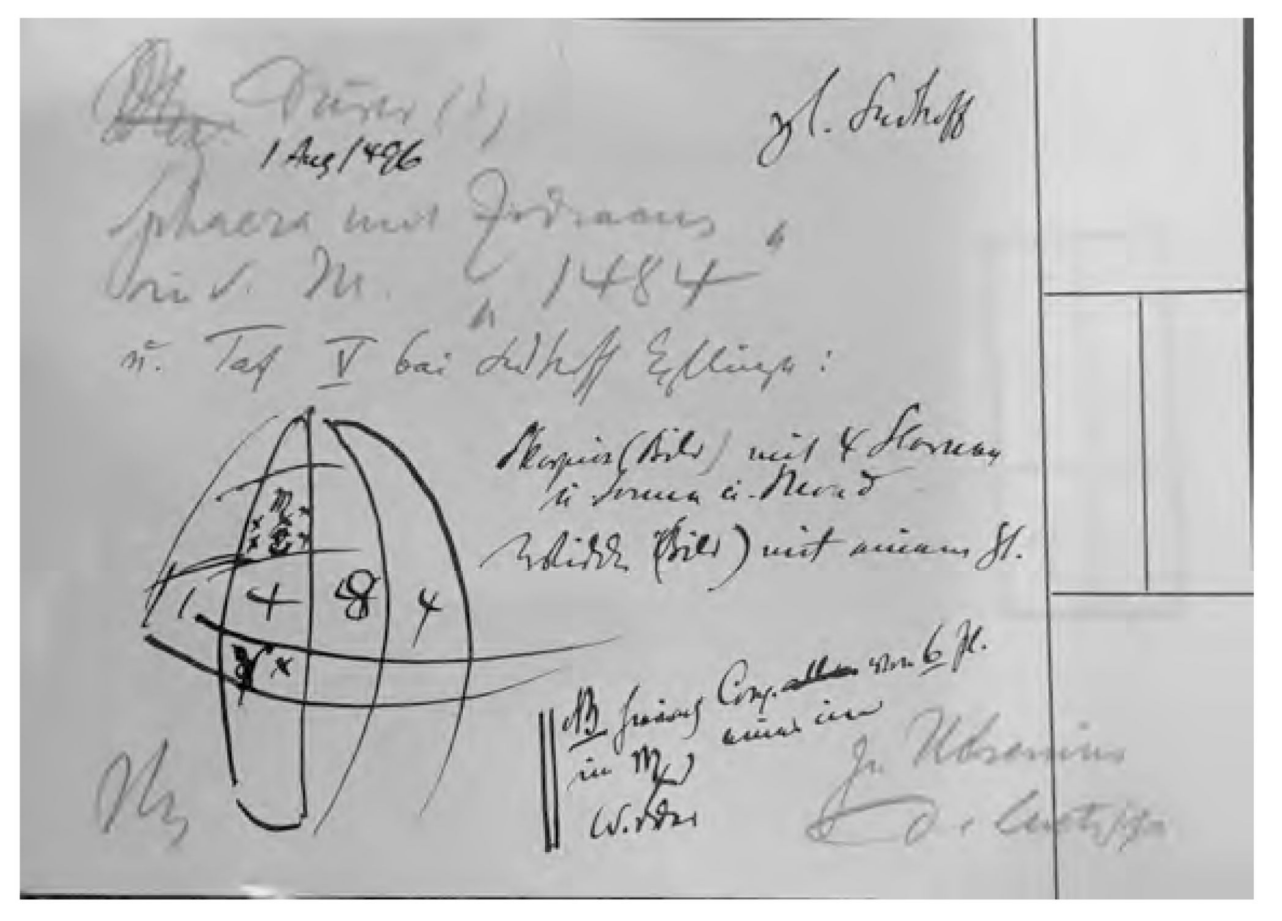
Warburg’s index card sketch reproducing the celestial sphere in Albrecht Dürer, *The Syphilitic Man* (Nürnberg: Hans Meier, 1496) from Karl Sudhoff, *Graphische und typographische Erstlinge der Syphilisliteratur aus den Jahren 1495 und 1496* (München: Carl Kühn, 1912), Tafel V. Warburg Institute Archive, Zettelkästen 29, 016191. Copyright: The Warburg Institute.

**Fig. 9:**
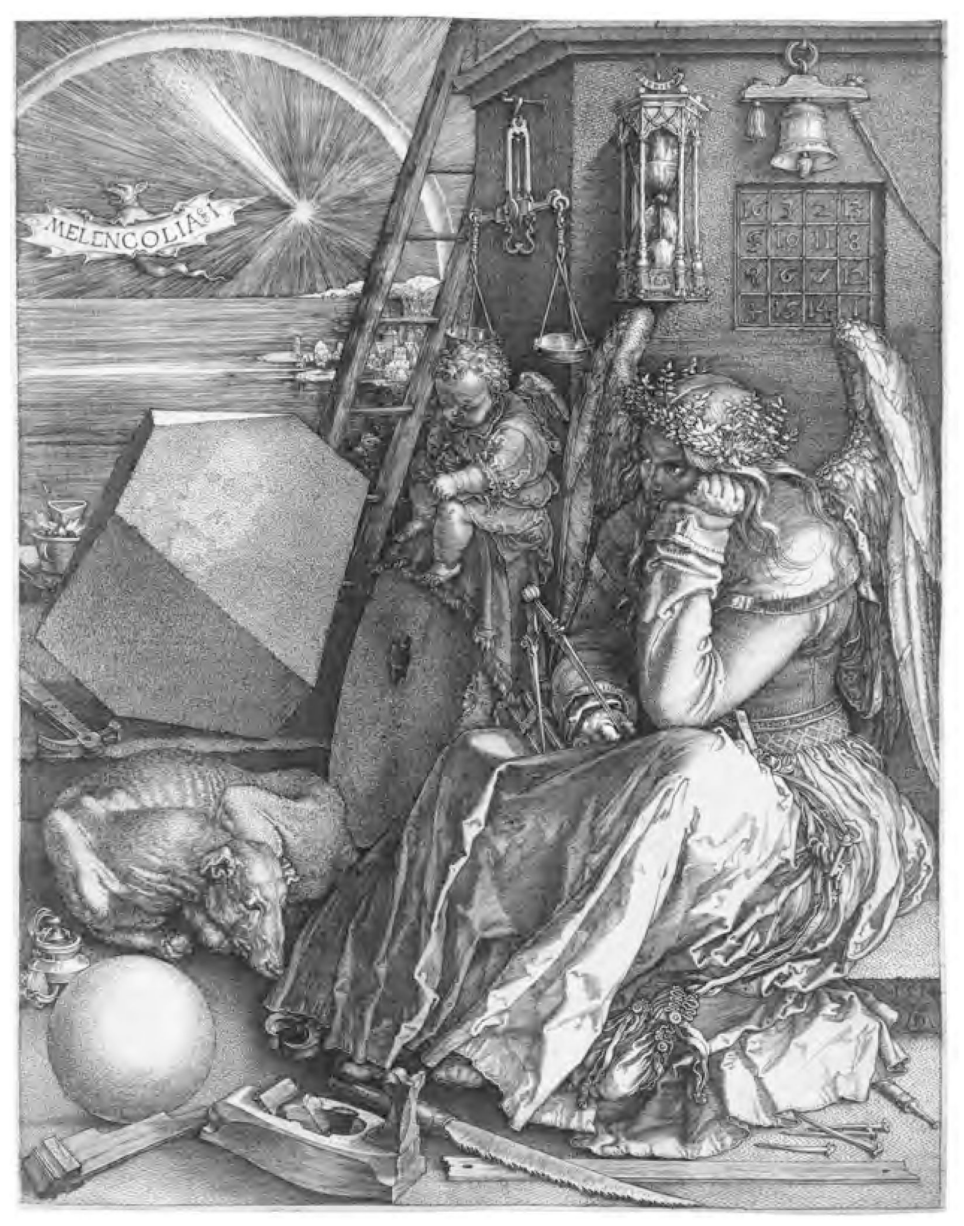
Albrecht Dürer, *Melencolia I* (1514). Wikimedia Commons, Public Domain.

**Fig. 10:**
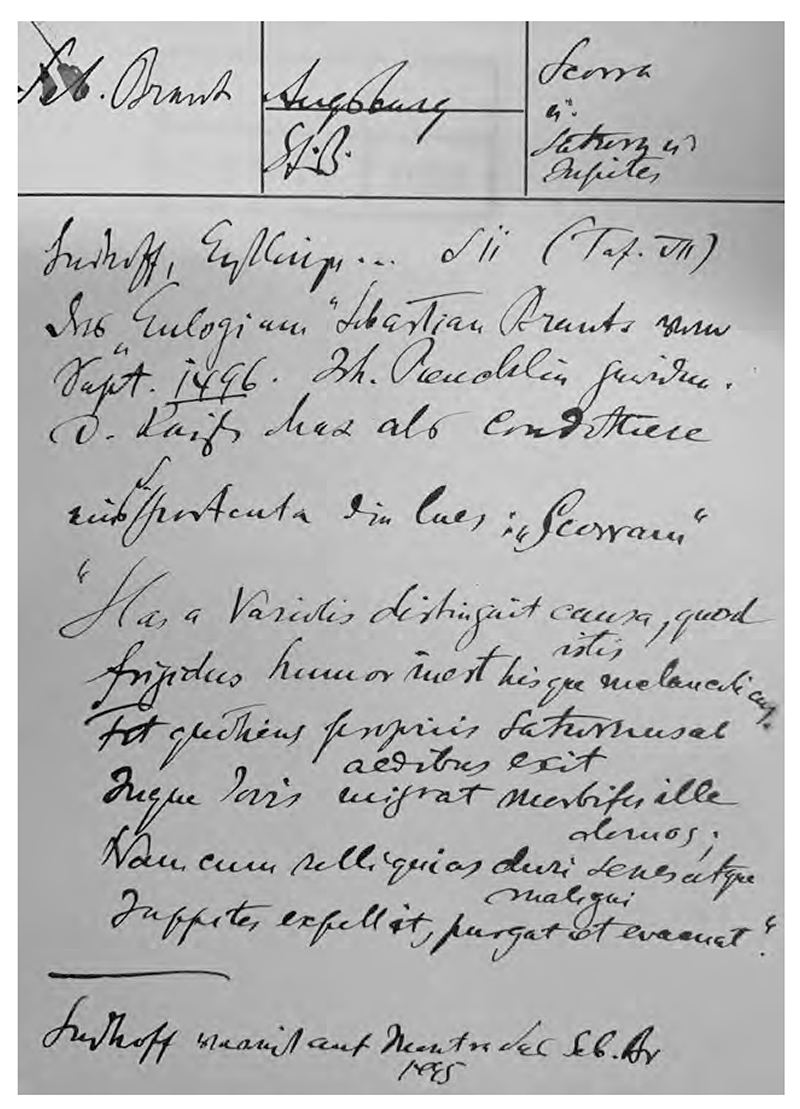
Warburg’s adaptation of a poem by Sebastian Brant on the iatromathematical cure of melancholy, from Karl Sudhoff, *Graphische und typographische Erstlinge der Syphilisliteratur aus den Jahren 1495 und 1496* (München: Carl Kühn, 1912), Tafel VII. Warburg Institute Archive, Zettelkästen 29, 016063. Copyright: The Warburg Institute.

## Footnotes

1 Letter from Fritz Saxl to Aby Warburg, 14 January 1927. This encounter was also described in Dorothea McEwan, *Fritz Saxl - Eine Biografie* (Böhlau, 2013), 111–13. All correspondences quoted are from Warburg Institute Archive, General Correspondence. All translations by the author unless otherwise noted.

2 “Diese Sammlungen sind eigentlich das Interessanteste und Anregendste, was ich seit langem gesehen habe,” Saxl to Warburg, 14 January 1927.

3 For an overview of recent scholarship on Warburg’s legacy, see David L. Marshall, “A Thousand Warburgs,” *Journal of the History of Ideas* 78, no. 4 (2017): 645–64.

4 Aby Warburg, *BAND II 2 Bilderreihen und Ausstellungen*, ed. Uwe Fleckner and Isabella Woldt (Berlin: De Gruyter, 2012).

5 Aby Warburg, “Reformatorische Weissagung in Wort and Bild,” Verein für Hamburgische Geschichte, 12 November 1917. Warburg gave three lectures concerning pagan prophecy in Luther’s age, the final version published as Aby Warburg, *Heidnisch-antike Weissagung in Wort und Bild zu Luthers Zeiten* (Heidelberg: Carl Winters Universitätsbuchhandlung, 1920).

6 Sudhoff to Warburg, 12 March 1916.

7 Sander L. Gilman, *Picturing Health and Illness: Images of Identity and Difference* (Baltimore: Johns Hopkins University Press, 1995), 24–30; Gundolf Keil, “Sudhoff and Medical History,” *Scientiarum Historia: Tijdschrift Voor de Geschiedenis van de Wetenschappen En de Geneeskunde* 29, no. 1 (2003): 87–88.

8 Vivian Nutton, “In the Shadow of Sudhoff: German Medical History in the Weimar Republic,” *Medical History* 49, no. 4 (2005): 521–24; Thomas Rütten, “Karl Sudhoff and ‘the Fall’ of German Medical History,” in *Locating Medical History: The Stories and Their Meanings*, ed. Frank Huisman and John Harley Warner (Baltimore: Johns Hopkins University Press, 2006), 96–100; Dominik Gross and Werner Friedrich Kümmel, “Karl Sudhoff (1853–1938) und der Nationalsozialismus,” *Sudhoffs Archiv* 100, no. 1 (2016): 2–22.

9 On Warburg and Weber, see Michael Diers, ed., *Warburg Aus Briefen: Kommentare Zu Den Kopierbüchern Der Jahre 1905–1918* (Weinheim: VCH Acta Humanoria, 1991), 94–97; Charlotte Schoell-Glass, *Aby Warburg and Anti-Semitism: Political Perspectives on Images and Culture* (Detroit: Wayne State University Press, 2008), 67–71.

10 Jane O. Newman, “Enchantment in Times of War: Aby Warburg, Walter Benjamin, and the Secularization Thesis,” *Representations* 105, no. 1 (February 2009): 133–67; Mario Wimmer, “The Afterlives of Scholarship: Warburg and Cassirer,” *History of Humanities* 2, no. 1 (2017): 245–70.

11 Dorinda Outram, “The Enlightenment Our Contemporary,” in *The Sciences in Enlightened Europe*, ed. William Clark, Jan Golinski, and Simon Schaffer (Chicago: University of Chicago Press, 1999), 32–40.

12 On Warburg’s influence in recent historiography of Reformation, see Brian P. Copenhaver, *Magic in Western Culture: From Antiquity to the Enlightenment* (Cambridge: Cambridge University Press, 2015), section I.2.6, “Warburg”; Robin B. Barnes, Astrology and Reformation (Oxford University Press, 2016), ch. 1. Also see Anthony Grafton, “Starry Messengers: Recent Work in the History of Western Astrology,” *Perspectives on Science* 8, no. 1 (2000): 70–83.

13 Frances Yates, *The Occult Philosophy in the Elizabethan Age* (London: Routledge & Kegan Paul, 1979), Charles Webster, *From Paracelsus to Newton: Magic and the Making of Modern Science* (Cambridge: Cambridge University Press, 1982). Also see Stanley Jeyaraja Tambiah, *Magic, Science, Religion, and the Scope of Rationality* (Cambridge: Cambridge University Press, 1990).

14 Elizabeth Fee and Theodore M. Brown, eds., *Making Medical History: The Life and Times of Henry E. Sigerist* (Baltimore: Johns Hopkins University Press, 1997); Susan M. Reverby and David Rosner, “‘Beyond the Great Doctors’ Revisited: A Generation of the ‘New’ Social History of Medicine,” in *Locating Medical History*, 167–93.

15 Ernst H. Gombrich, *Aby Warburg: An Intellectual Biography* (London: Phaidon, 1986).

16 Warburg to Mary Hertz, 15 September 1892. On Warburg’s response to this epidemic see Steffen Haug and Johannes von Müller, “Tragic Loss and Monumental Legacy: Warburg on Epidemics,” Mnemosyne: The Warburg Institute Blog, The Warburg Institute, accessed August 5, 2022, https://warburg.sas.ac.uk/blog/warburg-epidemics.

17 Karl Sudhoff, “Aus Meiner Arbeit,” *Archiv für Geschichte der Medizin* 21, no. 1–4 (1929): 342–43.

18 Henry E. Sigerist, “Karl Sudhoff, 1853–1938,” *Bulletin of the History of Medicine* 7, no. 7 (1939): 801–4. For Sudhoff’s works during this time, see Grethe Herbrand, “Systematisches Verzeichnis Der Arbeiten Karl Sudhoffs,” *Sudhoffs Archiv Für Geschichte Der Medizin Und Der Naturwissenschaften* 27, no. 1/2 (1934): 131–86; Grete Hochmuth, “Systematisches Verzeichnis Der Arbeiten Karl Sudhoffs: Nachtrag Für Die Jahre 1898–1933: Zu: Sudhoffs Archiv 27 (1934): 131–86,” *Sudhoffs Archiv Für Geschichte Der Medizin Und Der Naturwissenschaften* 32, no. 4/5 (1939): 279–84.

19 Sudhoff, “Aus Meiner Arbeit,” 358–60; Sigerist, 802.

20 Andreas Frewer et al., *Die Institutionalisierung der Medizinhistoriographie: Entwicklungslinien vom 19. ins 20. Jahrhundert* (Stuttgart: Franz Steiner, 2001); Christopher S. Wood, *A History of Art History* (Princeton, NJ: Princeton University Press, 2019), 288–91.

21 Andreas Frewer, “Biographie und Begründung der akademischen Medizingeschichte: Karl Sudhoffund die Kernphase der Institutionalisierung 1896–1906,” in *Die Institutionalisierung der Medizinhistoriographie*, 103–26.

22 Claudia Stein, “Divining and Knowing: Karl Sudhoff’s Historical Method,” *Bulletin of the History of Medicine* 87, no. 2 (June 26, 2013): 198–224.

23 “Mich interessiert als Medizinhistoriker zum Arzt des Stelzen selbst, um der ‘Prothese’ willen … ich denke der Fachhistoriker darf auch den Generalhistoriker der Kunst einmal auf Paralleler Hinreisen zu Forschungslinien vor denen erweise, dass der Historiker der Kunst ihnen Ergebnisse zur entlocken vermag, die direkt in seine Studiengänge hin ein zu wirke vermögen,” Sudhoff to Warburg, 12 March 1916. Also see Sudhoff, “Aus Meiner Arbeit,” 364.

24 Karl Sudhoff, *Beiträge zur Geschichte der Chirurgie im Mittelalter Graphische und textliche Untersuchungen in mittelalterlichen Handschriften*, Heft 10, Studien zur Geschichte der Medizin (Leipzig, 1914).

25 Warburg Institute Library, FEI 695.

26 Matthew Vollgraff, “Before the Atlas: Exhibiting Cultural History in Wilhelmine Germany,” public lecture at Haus der Kulturen der Welt, 3 November 2020, accessed 10 December 2021, https://www.youtube.com/watch?v=804l2JS0chg. For Sudhoff’s contribution see *Archiv für Buchgewerbe*, Band 51, Heft 1 (Leipzig: Verlag des Deutschen Buchgewerbevereins, 1914), 41–46; Ingrid Kästner, “Im Dienste Der Wissenschaft: Drei Jahrtausende Wissenschaftliche Grafik in Der Halle Der Kultur,” in *Die Welt in Leipzig: BUGRA 1914*, ed. Ernst Fischer and Stephanie Jacobs (Hamburg: Maximilian-Gesellschaft e.V, 2014).

27 Hans Christian Hönes, “Warburg’s Positivism: Confessions of a Truffle Pig,” *Oxford Art Journal* 41, no. 3 (December 1, 2018): 361–79.

28 Saxl to Warburg, 14 January 1927.

29 Karl Sudhoff, “Malerei und Geschichte der Medizin,” in Eugen Holländer, *Katalog zur ausstellung der geschichte der medizin in kunst und kunsthandwerk: Zur eröffnung des Kaiserin Friedrich-Hauses 1. märz 1906* (Stuttgart: Ferdinand Enke, 1906), 27–33. Translation adapted from Karl Sudhoff, *Essays in the History of Medicine*, trans. Fielding H. Garrison (New York: Medical Life Press, 1926), 313–17.

30 Kathryn Brush, “Aby Warburg and the Cultural Historian Lamprecht,” in *Art History as Cultural History: Warburg*’*s Projects*, ed. Richard Woodfield (London: Routledge, 2001), 65–91; Dieter Wuttke, “Aby M. Warburgs Kulturwissenschaft,” 1993, 1–30.

31 Stein, *Divining and Knowing*.

32 Steffen Haug and Johannes von Müller, “Aby Warburg and the Foundations of Image Studies,” in *The Palgrave Handbook of Image Studies*, ed. Krešimir Purgar (Cham: Springer International Publishing, 2021), 131–46; Horst Bredekamp, “A Neglected Tradition? Art History as Bildwissenschaft,” *Critical Inquiry* 29, no. 3 (2003): 418–28.

33 Saxl to Warburg, 14 January 1927.

34 Lorraine Daston and Peter Galison, *Objectivity* (New York: Zone Books, 2007).

35 Karl Sudhoff, “Photographie Oder Zeichnung?,” *Wochenschrift Für Klassische Philologie* 28, no. 10 (März 1911): 279.

36 Michael Diers, “Atlas und Mnemosyne: Von der Praxis der Bildtheorie bei Aby Warburg,” in *Bildtheorien: Anthropologische und Kulturelle Grundlagen des Visualistic Turn* (Frankfurt: Suhrkamp, 2009), 181–213; Katia Mazzucco, “(Photographic) Subject-Matter: Fritz Saxl Indexing Mnemosyne—A Stratigraphy of the Warburg Institute Photographic Collection’s System,” *Visual Resources* 30, no. 3 (2014): 201–21.

37 Franz Boll to Warburg, 22 February 1916. Sudhoff to Warburg, 12 March 1916.

38 Warburg to Sudhoff, 20 March 1916.

39 Warburg, *Heidnisch-antike Weissagung*, 24–34.

40 Lucia Ruprecht, “Gestures between Symptom and Symbol in Aby Warburg and Sigmund Freud,” in *Gestural Imaginaries* (New York: Oxford University Press, 2019), 125–50.

41 Warburg’s note for the lecture on serpent ritual. Quoted from Gombrich, *Intellectual Biography*, 223.

42 Jacob Burckhardt, *Reflections on History* (Indianapolis: Liberty Classics, 1979): 34; Martin Wiklund and Pedro Spinola Pereira Caldas, “Introduction: History as Diagnosis,” *Tempo* 25, no. 3 (December 2019): 667–72.

43 Georges Didi-Huberman, “Dialektik Des Monstrums: Aby Warburg and the Symptom Paradigm,” *Art History* 24, no. 5 (2001): 621–45.

44 Carlo Ginzburg, “Clues: Roots of a Scientific Paradigm,” *Theory and Society* 7, no. 3 (1979): 273–88.

45 “Eine Eigenschaft vor allen ist dem bildenden Künstlerund dem wahren Arzte gemeinsam und in ihrer vollsten Ausbildung eigentlich nur diesen beiden gegeben, das wirklichkeitsoffene Auge, der alles erfassende Blick, die große Kunst des Sehens, die ihn zwingt, alle Hüllenzu durchdringen, unter der Schale den Kern, unter der Oberfläche das spielend sich wegende Leben zu sehen,” Sudhoff, *Malerei und Geschichte der Medizin*. Translation modified from Sudhoff, *Essays*, 313–17.

46 Claudia Wedepohl, “Pathos—Polarität—Distanz—Denkraum: Eine archivarische Spurensuche,” in *Warburgs Denkraum: Formen, Motive, Materialien*, ed. Martin Treml, Sabine Flach, and Pablo Schneider (Munich: Wilhelm Fink, 2014), 15–49. Erwin Panofsky, *Studies in Iconology: Humanistic Themes in the Art of the Renaissance* (New York: Harper & Row, 1962), 3; “Iconography and Iconology,” in *Meaning in the Visual Arts: Papers in and on Art History* (Garden City, NY: Doubleday, 1955), 30.

47 Emily J. Levine, *Hamburg, Dreamland of Humanists: Warburg, Cassirer, Panofsky, and the Hamburg School* (Chicago: University of Chicago Press, 2013), Introduction; Peter Burke, *Eyewitnessing: The Uses of Images as Historical Evidence* (London: Reaktion Books, Limited, 2014).

48 Sudhoff, *Malerei und Geschichte der Medizin*, translation from Sudhoff, *Essays*, 313–17.

49 Michel Foucault, *The Birth of the Clinic: An Archaeology of Medical Perception* (London: Routledge, 2009).

50 Sudhoff, “Aus Meiner Arbeit,” 374. Claudia Stein, “Organising the History of Hygiene at the Internationale Hygiene-Ausstellung in Dresden in 1911,” *NTM Zeitschrift für Geschichte der Wissenschaften, Technik und Medizin* 21, no. 4 (December 1, 2013): 355–87.

51 Thomas Steller, *Das Neue Wissen vom Menschen. Entstehung und Entwicklung des Deutschen Hygiene-Museums in Dresden 1900–1931: Zur Popularisierung eines modernen Menschenbildes* (Munich: GRIN Verlag, 2010).

52 Otto Neustätter to Warburg, 2 May 1910.

53 Warburg to Neustätter, 1 June 1910.

54 Iatromathematik means “the medical implications of astrology, i.e., the recognition of a nosological predisposition of patients or a prognosis of current illnesses, connected with prevention or therapy, depending on the case,” Alain Touwaide, “Iatromathematics,” Brill’s New Pauly (German: “Iatromathematik,” Der Neue Pauly), 2006. Other interchangeable terminologies used in Warburg and Neustätter’s correspondences include Iatrochemie, Iatroastrologie, Iatromystik, etc., now mostly replaced by “astrological medicine.” See Karl Rothschuh, “Zur Terminologie in der Medizingeschichtsschreibung, insbesondere bei den Iatrowissenschaften,” *Sudhoffs Archiv* 61, no. 1 (1977): 5–7.

55 Warburg to Neustätter, 26 January 1911.

56 Karl Sudhoff, *Iatromathematiker vornehmlich im 15. und 16. Jahrhundert* (Breslau: J.U. Kern & M. Müller, 1902). Fielding H. Garrison, “Biographical Sketch,” in Sudhoff, *Essays*, 17.

57 Neustätter to Warburg, 3 June 1910.

58 Warburg to Neustätter, 6 May 1911.

59 Warburg to Neustätter, between 17 and 22 June 1910. According to Stein, *Organising the History of Hygiene*, Sudhoff worked from Leipzig for the Dresden exhibition and only came to Dresden occasionally.

60 Neustätter to Warburg, 21 January 1911; Warburg to Neustätter, 26 January 1911.

61 “diese ganze Periode eine Hemmung der Hygiene bedeutete,” Neustätter to Warburg, 23 January 1910.

62 Sudhoff, *Iatromathematiker*, 5.

63 Karl Sudhoff, “Hohenheim Und Die Medizinische Astrologie (Astrologische Medizin),” *Verhandlungen der Gesellschaft deutscher Naturforscher und Ärzte* 76, no. 2 (1904): 78–79.

64 Sudhoff, “Aus Meiner Arbeit,” 339. Heinrich Haeser, *Lehrbuch Der Geschichte Der Medicin Und Der Epidemischen Krankheiten*, 3. Bearbeitung (Jena: Verlag von Hermann Dufft, 1875–1882). Sudhoff’s personal copy is kept at Bayerische Staatbibliothek, Bibl. Sud. 2857-1 (vol. 1), Bibl. Sud. 2378-3 (vol. 3).

65 Sudhoff, *Iatromathematiker*, 8.

66 Karl Sudhoff, *Graphische und typographische Erstlinge der Syphilisliteratur aus den Jahren 1495 und 1496* (München: Carl Kühn, 1912), 8. Sudhoff, *Malerei und Geschichte der Medizin*, 28. Translation from Sudhoff, *Essays*, 49.

67 Sudhoff, *Essays*, 47.

68 Heinz-Peter Schmiedebach, “Bildung in a Scientific Age,” in *Locating Medical History*, 74–94.

69 Neustätter to Warburg, 24 June 1910; Warburg to Neustätter, 6 May and 12 August 1911. For the wrong catalog entry see *Historische Abteilung Mit Ethnographischer Unterabteilung. I: Historische Abteilung*. 2. verb. und illustrierte Aufl. (Dresden: Internationalen Hygiene-Ausstellung, 1911), 399, item 14331.

70 Warburg to Neustätter, 12 May 1911.

71 Warburg to Neustätter, 12 August 1911.

72 Neustädter to Warburg, 23 November 1910.

73 Sudhoff, “Aus Meiner Arbeit,” 375.

74 Vollgraff, *Before the Atlas*.

75 Christopher D. Johnson, *Memory, Metaphor, and Aby Warburg’s “Atlas of Images”* (Ithaca, NY: Cornell University Press, 2012), introduction and 194–229.

76 Aby Warburg, *Tagebuch der Kulturwissenschaftlichen Bibliothek*, ed. Karen Michels and Charlotte Schoell-Glass (Berlin: De Gruyter, 2001), 488.

77 Stein, *Divining and Knowing*, 209.

78 Karl Sudhoff and Charles Singer, *The Earliest Printed Literature on Syphilis; Being Ten Tractates from the Years 1495–1498, In Complete Facsimile, with an Introduction and Other Accessory Material* (Florence: Lier, 1925), 10.

79 Sudhoff, *Syphilisliteratur*, 8. Also see Karl Sudhoff, *Aus der Frühgeschichte der Syphilis; Handschriften- und Inkunabelstudien, epidemiologische Untersuchung und kritische Gänge* (Leipzig: J. A. Barth, 1912).

80 Paulus von Middelburg, *Prognostica Ad Viginti Annos Duratura* (Antwerp: Gerard Leeu, 1484).

81 Sudhoff, *Frühgeschichte*, 159–61.

82 Warburg to Sudhoff, 9 May 1917.

83 *Heidnisch-antike Weissagung*, 56.

84 *Heidnisch-antike Weissagung*, 38. Johannes Lichtenberger, *Pronosticatio in latino* (Heidelberg: Vocabularius, 1488). Warburg owned a later German translation of this book, *Die Weissagunge Johannis Lichtenbergers Deudsch, Zugerichtmit Vleys* (Wittenberg: Getruckt durch Hans Lufft, 1527). Warburg Library, FAH 5527.

85 “Ich kann Ihnen aber schon verraten, dass sich sehr merkwürdige Beziehungen zwischen Middelburg und Lichtenberger nachweisen lassen, die für die Bewertung der früheren Prognostica unter ganz allgemeinen kulturwissenschaftlichen Gesichtspunkten erwünschte und unbestreitbare Aufklärungen ergeben,” Warburg to Sudhoff, 16 May 1917.

86 *Heidnisch-antike Weissagung*, 81–86.

87 Webster, *From Paracelsus to Newton*, 5–22; Hartmut Rudolph, “Einige Gesichtspunkte zum Thema ‘Paracelsus und Luther,’” *Archiv für Reformationsgeschichte* Heft 72 (December 1, 1981): 34–54.

88 Colin Eisler, “Who Is Dürer’s ‘Syphilitic Man?,’” *Perspectives in Biology and Medicine* 52, no. 1 (2009): 48–60.

89 Sudhoff, *Syphilisliteratur*, 11. Warburg to Sudhoff, 9 May 1917.

90 Ernst Cassirer, *Individuum und Kosmos in der Philosophie der Renaissance* (Wiesbaden: Vieweg+Teubner Verlag, 1927), 111.

91 Yates, *Occult Philosophy in the Elizabethan Age*, 13.

92 This term was first used in Robert S. Westman, “Magical Reform and Astronomical Reform: The Yates Thesis Reconsidered,” in *Hermeticism and the Scientific Revolution: Papers Read at a Clark Library Seminar, March 9, 1974* (William Andrews Clark Memorial Library, 1977), 3–91. For debates concerning the “Yates Thesis” see Wouter J. Hanegraaff, “Beyond the Yates Paradigm: The Study of Western Esotericism Between Counterculture and New Complexity,” *Aries* 1, no. 1 (2001): 5–37.

93 Benjamin Lazier, “Overcoming Gnosticism: Hans Jonas, Hans Blumenberg, and the Legitimacy of the Natural World,” *Journal of the History of Ideas* 64, no. 4 (2003): 619–37.

94 *Heidnisch-antike Weissagung*, 56. Translation from *The Renewal of Pagan Antiquity*, 650. Georges Didi-Huberman, “The Surviving Image: Aby Warburg and Tylorian Anthropology,” *Oxford Art Journal* 25, no. 1 (2002): 61–69.

95 Horst Bredekamp and Claudia Wedepohl, *Warburg, Cassirer und Einstein im Gespräch: Kepler Als Schlüssel der Moderne* (Berlin: Wagenbach, 2015).

96 Spyros Papapetros, “‘Against Nature’: Warburg and Regressive Evolution,” in *Aby Warburg und die Natur, Epistemik, Ästhetik, Kulturtheorie*, ed. Frank Fehrenbach and Cornelia Zumbusch (Berlin: De Gruyter, 2019), 149–216.

97 Lauren Kassell, “The Economy of Magic in Early Modern England,” in *The Practice of Reform in Health, Medicine, and Science, 1500–2000: Essays for Charles Webster*, ed. Margaret Pelling and Scott Mandelbrote (London: Routledge, 2017), 43–57.

98 Friedrich Nietzsche, *Vom Nutzen und Nachteil der Historie für das Leben* (Fritzsch, 1874).

99 Bruno Latour, *We Have Never Been Modern* (Cambridge, MA: Harvard University Press, 1993), 74.

100 Jane O. Newman, “Luther’s Birthday: Aby Warburg, Albrecht Dürer, and Early Modern Media in the Age of Modern War,” *Daphnis* 37, no. 1–2 (2008): 79–110; Gottfried Korff, “Im Zeichen des Saturn. Vorläufige Notizen zu Warburgs Aberglaubensforschung im Ersten Weltkrieg,” in *Kasten 117: Aby Warburg und der Aberglaube im Ersten Weltkrieg*, ed. Gottfried Korff (Tübingen: Tübinger Vereinigung für Volkskunde, 2007), 181–213.

101 Sudhoff, *Essays*, 50.

102 Weber to Tönnies, 2 March 1999. Quoted in Jason Josephson-Storm, *The Myth of Disenchantment: Magic, Modernity, and the Birth of the Human Sciences* (Chicago: University of Chicago Press, 2017), 288.

103 Wouter J. Hanegraaff, *Esotericism and the Academy: Rejected Knowledge in Western Culture* (Cambridge: Cambridge University Press, 2012), ch. 4; Egil Asprem, *The Problem of Disenchantment: Scientific Naturalism and Esoteric Discourse 1900–1939* (Boston: Brill, 2014).

104 Jason Crawford, “The Trouble with Re-Enchantment,” *Los Angeles Review of Books*, September 7, 2020. Accessed 10 June 2022, https://lareviewofbooks.org/article/the-trouble-with-re-enchantment/; J. C. D. Clark, “The Re-Enchantment of the World? Religion and Monarchy in Eighteenth-Century Europe,” in *Monarchy and Religion: The Transformation of Royal Culture in Eighteenth-Century Europe*, ed. Michael Schaich (Oxford: Oxford University Press, 2007), 41–75.

105 Alexandra Walsham, “The Reformation and ‘The Disenchantment of the World’ Reassessed,” *The Historical Journal* 51, no. 2 (June 2008): 497–528; Richard Jenkins, “Disenchantment, Enchantment and Re-Enchantment: Max Weber at the Millennium,” *Max Weber Studies* 1, no. 1 (2000): 11–32.

